# One-Step Synthesis of Eu^3+^-Modified Cellulose Acetate Film and Light Conversion Mechanism

**DOI:** 10.3390/polym13010113

**Published:** 2020-12-30

**Authors:** Zhihui Zhang, Zhengdong Zhao, Yujia Lu, Di Wang, Chengyu Wang, Jian Li

**Affiliations:** 1 Engineering Research Center of Advanced Wooden Materials (Ministry of Education), Northeast Forestry University, Harbin 150040, China; zzh13251613906@nefu.edu.cn (Z.Z.); Zhaozd6866@163.com (Z.Z.); luyujia1999@163.com (Y.L.); wangcy@nefu.edu.cn (C.W.); nefujianli@163.com (J.L.); 2 Key Laboratory of Bio-based Material Science and Technology (Ministry of Education), Northeast Forestry University, Harbin 150040, China; 3 Collage of Material Science & Engineering, Northeast Forestry University, Harbin 150040, China

**Keywords:** light conversion film, cellulose acetate, europium, sensitization

## Abstract

A CA-Eu(III) complex was synthesized by the coordination reaction of cellulose acetate (CA) and Eu^3+^ to obtain a CA-Eu light conversion film. This product was then doped with Tb(III) to sensitize the luminescence of Eu^3+^, which could functionalize the CA film. FTIR and XPS showed that the oxygen atoms in C=O, C–O (O=C–O), and O–H were involved in the complexation with Eu^3+^ and formed a Eu–O bond. SEM revealed that Eu^3+^ filled in the pores of the CA film. By changing the experimental conditions, the best fluorescence performance was obtained at the CA: Eu^3+^ ratio of 3:1 with a reaction time of 65 min. The energy transfer between Tb^3+^–Eu^3+^ could be realized by doping Tb^3+^ to enhance the luminescence of Eu^3+^. The best fluorescence performance of the CA-Eu-Tb light conversion film was at a Eu^3+^:Tb^3+^ ratio of 3:1. Compared with the CA film, the light conversion film has high transparency, high tensile strength, and good flexibility. It can convert the ultraviolet light harmful to plants into red light that is beneficial to photosynthesis. This offers high efficiency and environmental protection in the field of agricultural films.

## 1. Introduction

Agricultural light conversion films achieve light conversion by adding light conversion agents, thereby changing the light quality. The absorption spectrum of chlorophyll is in the blue-violet light region (400–500 nm) and the red-orange light region (600–680 nm). The agricultural light conversion film blocked the harmful ultraviolet light and converted it into red-orange light, which promoted the photosynthesis of plants and improved the use of light, thereby increasing the yield of crops [1].

The light conversion agents used in agricultural films can be divided into rare earth organic ligand light conversion agents, rare earth inorganic light conversion agents, and fluorescent dyes according to the light conversion principles and the general category of elements. To explore highly efficient yellow-green light conversion agents used for agricultural film, Yu et al. [2] prepared six bay-substituted perylene diimides and their polyvinyl chloride-doped films. Qi et al. [3] explored a new type of light conversion film based on aggregation-induced emission (AIE). Gong et al. [4] obtained a series of multicolor Si NPs powders with fluorescence colors from blue to red by a facile high temperature calcination strategy for the first time and the carboxymethyl cellulose (CMC)/Si NPs powders obtained by calcining under air atmosphere for 2 min (SSF-2) (CMC/SSF-2) films with strong UV absorption, good transparency, and high tensile strength have been obtained with great potential in the field of agricultural planting. However, most mulching films were made of polyethylene, causing white pollution when they remained in the soil after use [5]. Therefore, the use of degradable mulch is an excellent way to solve the pollution problem [6].

Yu [7] et al. researched and synthesized a polylactic acid/polybutylene terephthalate/rare earth complex biodegradable light conversion agricultural film. Wang et al. [8] synthesized a rare earth europium (III) complex with α-thenoyltrifluoroacetone and triphenylphosphine oxide (Eu(TTA)_3_(TPPO)_2_) and then blended this product with polylactide (PLA) and poly(butylene adipate-co-terephthalate) (PBAT) to prepare a biodegradable and efficient blue-violet light conversion agricultural film. They further explored this for environmental pollution. In addition, some light conversion agents had poor dispersibility and compatibility with the base resin of the greenhouse film, which may deteriorate the processing performance and even affect the mechanical properties of the agricultural film during the production of the greenhouse film. To solve these problems, research into rare earth polymer complex fluorescent materials is needed.

The polymer ligands of this type of material had good compatibility with the base resin, and the central ions usually included Eu^3+^, Sm^3+^, and Er^3+^ [9]. Cellulose is the most abundant renewable resource on the earth, and its functionalization has always been a hot research topic. Cellulose is renewable with large reserves and easy biodegradability. It can be used to construct various functional polymer materials, and cellulose acetate (CA) is one of the most widely used cellulose derivative materials. This is a film-forming material with good performance—the molecular chain contained a large number of polar groups such as hydroxyl and acetyl groups, and the intermolecular forces are large as well [10,11].

Based on reference with the light conversion agricultural film of the predecessors, we used a rare earth ion Eu (III) with higher light conversion efficiency to modify CA. Here, CA-Eu (III) complexes were synthesized by the reaction of CA and Eu^3+^ to prepare a CA film modified with rare earth ions, and doped with Tb (III) to sensitize the luminescence of Eu (III) and functionalize the CA film. The luminescence performance was mainly studied to determine the best production conditions to improve the utilization rate of rare earths to reduce costs; this can be used in a wide range of agricultural films. The organic ligand CA in the films had a good absorption coefficient in the ultraviolet wavelength range. As long as the ligand could meet the requirements of energy transfer, it could solve the f-f prohibition of rare earth ions through ultraviolet absorption and transfer after coordination with the rare earth ions to enhance the luminous efficiency of rare earth (Antenna effect) [12]. Due to its high transparency, high tensile strength, good flexibility, and absorption of ultraviolet light to enhance visible light and biodegradable characteristics, it offers high efficiency and environmental protection in the field of agricultural films.

In addition, the product (casting liquid) of this experiment was the organic rare earth materials, which has the characteristics of low toxicity, high fluorescence chroma, and good oil solubility. Therefore, it could also be used for anti-counterfeiting identification, highlighter oils, and other directions.

## 2. Materials and Methods

### 2.1. Preparation of Ion-Modified Cellulose Acetate Light Conversion Film

First, cellulose acetate (CA, the degree of substitution (DS) = 2.45, acetyl 39.8 wt%, hydroxyl 3.5 wt%, Aladdin Bio-Chem Technology Co., Ltd., Shanghai, China) (2.6667 g, 1.0 mol) and europium nitrate hexahydrate (99.9%, Huawei Ruike Chemical Co., Ltd., Beijing, China) (2.2453 g, 5.03 × 10^−3^ mol) were dissolved separately in acetone (analytical grade, Fuyu Fine Chemical Co., Ltd., Tianjin, China) (20 mL for CA (in a single-mouthed flask) and 10 mL for Eu (in a beaker)) until they were completely dissolved. The sample was then poured into a single-mouthed flask and mixed evenly. CA in the mixed solution was 10 wt%, and the molar ratio of CA (structural units) to rare earth ions was 2:1. The mixing solution was stirred with magnetic stirrer at constant temperature (80 °C) under reflux for 35 min and cooled to room temperature, leading to a rare earth metal europium-modified cellulose acetate solution.

Control variables and change of other reaction conditions. Here, CA: Eu^3+^ ratios of 1:1, 2:1, 3:1, 4:1, and 5:1 were used. The doping molar ratio variable (Eu^3+:^ Tb^3+^ ratio of 1:1, 2:1, 3:1, 4:1, 5:1 and 1:1, 1:2, 1:3, 1:4, 1:5) and the reaction time variable (35, 45, 65, and 75 min) were also optimized. A series of ion-modified cellulose acetate solutions were then prepared under different reaction conditions.

The appropriate amount of ion-modified cellulose acetate solution was cast on a dry and clean glass plate, and the film was scraped with a SZQ type four-sided preparation device (AI Testing Instrument Co., Ltd., Taizhou, China); the thickness of the film was set to 1000 μm.

### 2.2. Structural Characterization and Performance Testing

The chemical groups of the films were detected by the Fourier-transform infrared (FTIR) spectroscopy (FTIR-650) (Gangdong Technology Co. Ltd., Tianjin, China): 4000–600 cm^−1^, 32 scans, and resolution of 4 cm^−1^. X-ray photoelectron spectroscopy (XPS, PHI5700 ULVCA-PHI, Chigasaki, Japan) was used to characterize the chemical structure of the films, and Casa XSP software was used to analyze the spectra.

Surface and cross-sectional morphology of the films were obtained by scanning electron microscope (SEM) (Apreo s, Thermo Fisher Scientific, Waltham, MA, USA). The films were immersed in liquid nitrogen for two minutes, and the cross-section samples were prepared by the brittle fracture method. The surface and the cross-section samples were sprayed with gold for observation on the experimental bench. The acceleration voltage was 5 kV.

The UV absorption and transmission spectra of the films were detected by TU-1950 UV-visible spectrophotometer (General Analysis General Instrument Co., Ltd., Beijing, China) from 200 to 700 nm.

The fluorescence spectroscopy (FL) was detected by HORIBA highly sensitive integrated FluoroMax-4 fluorescence spectrometer (HORIBA Scientific Instruments Division, Sunnyvale, CA, USA), and the scanning speed was 600 nm·min^−1^.

The mechanical properties were detected by CMT5504 electronic universal testing machine (New Sansi Metrology Technology Co., Ltd., Shenzhen, China). The test method was based on GB/T 1040.3-2006, and three sets of samples were tested in parallel. The flat, clean, and defect-free films were cut into 12 × 100 mm^2^ strips for testing. The test speed was set to 2.0 mm/min, and the nominal distance was 45 mm. The load displacement curve was obtained through tensile testing, and the tensile performance parameters of the films could then be obtained: tensile strength (σ_M_) and elongation at break (ε_B_).

The thermal stability of the films was obtained by thermogravimetric analyzer (TG 209 F1, Netzsch instruments Co. Ltd., Bavarian, Germany) under a nitrogen atmosphere. The test temperature range was 30 to 800 °C at a heating rate of 10 °C/min.

## 3. Results

### 3.1. Structure and Morphology Characterization of Films

**Figure 1:**
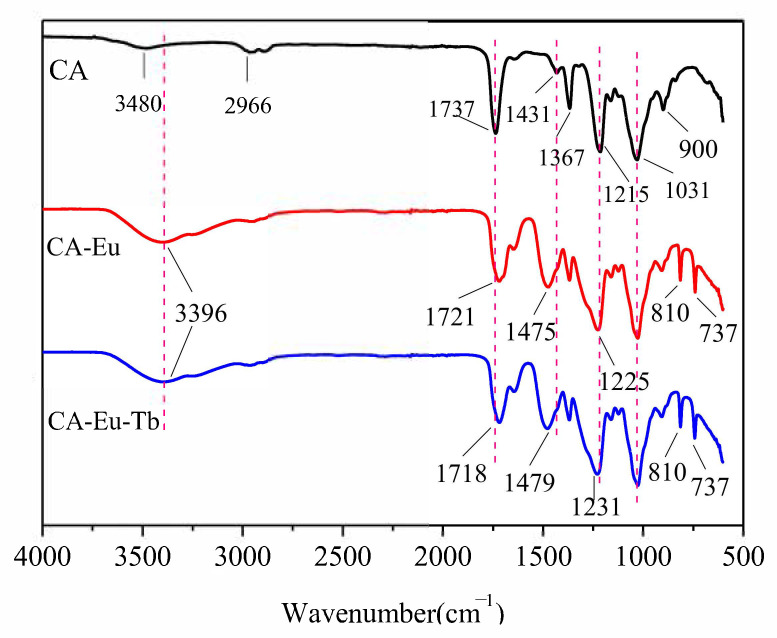
FT-IR spectra pure CA, CA-Eu (2:1), and CA-Eu-Tb (Eu: Tb = 1:1) films.

The infrared spectra of CA, CA–Eu (2:1), and CA–Eu–Tb (Eu:Tb = 1:1) films were shown in Figure 1. Of these, there was a wide peak at 3480 cm^−1^ in the pure CA film, which is attributed to the stretching vibration peak of O–H. However, the O–H group in the light conversion films red-shifted (the O–H had moved to 3396 cm^−1^) and the absorption peak was obviously broadened and stronger. This is because the hydroxyl group can be freed from the hydrogen bonds formed with other groups after the hydroxyl group on the CA molecular chain coordinated with Eu^3+^/Tb^3+^ [13]. The band observed at 2966 cm^−1^ belongs to the C–H stretching vibration peak [14]. The peaks at 1737 cm^−1^ and 1431 cm^−1^ correspond to the symmetric and antisymmetric stretching vibrations of –COO^−^ in CA, whereas both ν_s_ (–COO^−^) and ν_as_ (–COO^−^) had shifted in the light conversion films with a broadened peak shape, which initially indicated that Eu^3+^/Tb^3+^ coordinated with the O atom in the carbonyl group. The absorption peaks at 1031 cm^−1^ and 1215 cm^−1^ are attributed to the symmetrical stretching peak of C–O–C (there was basically no movement in the light conversion films) and the asymmetric stretching of C–O–C (blue shift to the position near 1225 cm^−1^ in the light conversion films) in the ester group [15], respectively. The peaks observed at 1367 cm^−1^ and 900 cm^−1^ are due to the characteristic absorption peaks of –CH_3_ [16] and the β-linked glucan structure [17]. The new vibration peaks appeared in the light conversion films at 810 cm^−1^ and 737 cm^−1^, which correspond to the infrared characteristic peaks of Eu–O and Tb–O [18] indicating the formation of coordination structures. Table S1 (see Supplementary Materials) summarizes the positions and changes of some absorption peaks of the films.

**Figure 2:**
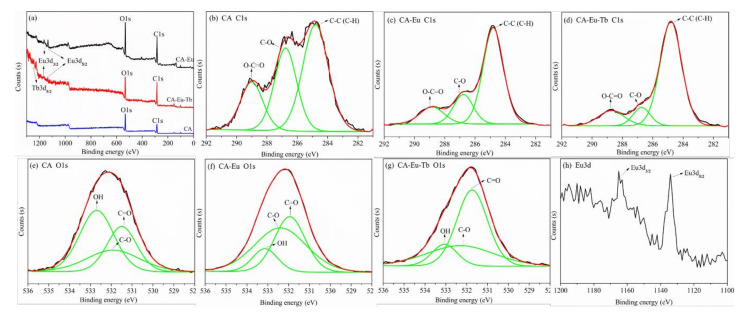
The XPS spectra of CA, CA–Eu and CA–Eu–Tb films. (**a**)—the survey spectrum, (**b**)—the C1s spectra for CA, (**c**)—CA–Eu, (**d**)—CA–Eu–Tb; (**e**)—the O1s spectra for CA, (**f**)—CA–Eu, (**g**)—CA–Eu–Tb, (**h**)—the Eu3d spectra.

To further explore the elements and structural composition of the light conversion films, XPS was performed to characterize the functional groups on the surface. An XPS survey spectrum was shown in Figure 2a. The main constituent elements of the pure CA film were C and O, and their contents were C (68.21 At%), and O (31.79 At%), respectively. In addition to the C and O elements contained in the light-converting films, Eu or Tb also appeared further indicating that Eu (III)/Tb (III) reacted with CA successfully and doped into the light-converting films.

**Figure 3:**
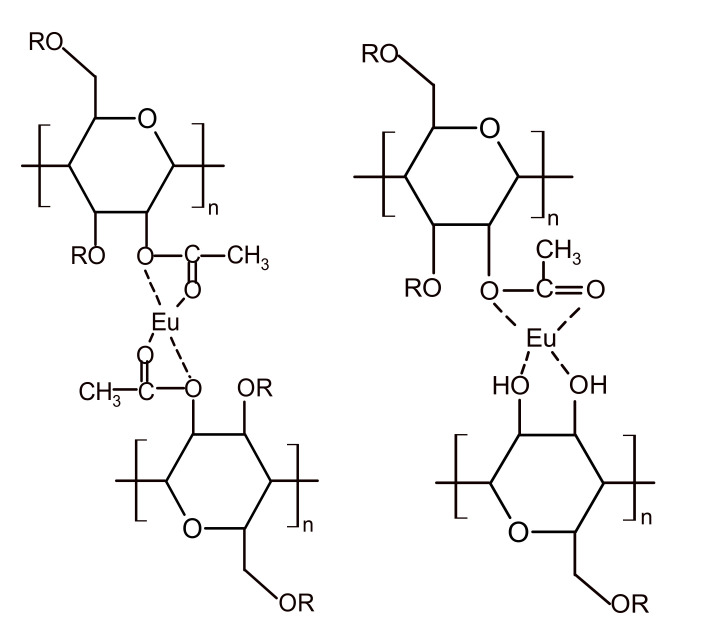
Schematic diagrams of the coordination structure between CA and Eu (III). (R = H or CH_3_CO–).

The C1s and O1s chemical bonds in the pure CA film, CA-Eu, and CA–Eu–Tb light conversion films were analyzed by peak fitting method. Figure 2b is the C1s spectrum of CA, containing three peaks positioned at 284.8, 286.774, and 289.002 eV, which were respectively assigned to C–C or C–H in the molecular chain skeleton, C–OH and C–O–C bond in the pyranose ring, and O=C–O in the acetyl group, respectively [19,20,21]. As shown in Figure 2c,d, the spectral shapes changed and the O–C=O bond of CA–Eu and CA–Eu–Tb light-converting films had moved 0.163 eV and 0.269 eV to the lower binding energy position compared with CA film, respectively. This is because the electron density around the C atom increased because of the coordination reaction accompanied by electron transfer; thus, its inner electron binding energy decreased inevitably. The O1s spectrum of CA was de-convoluted into three peaks positioned at 531.493, 531.907, and 532.693 eV; they belonged to the –OH, C–O, and carbonyl C=O bonds, respectively [22]. The three peaks in the CA–Eu film were located at 531.945, 532.393, and 533.131 eV, and these peaks in the CA–Eu–Tb film were located at 531.74, 532.297, and 533.06 eV. Compared with the CA film, the binding energy of O1s in the light conversion films all shifted to a higher position because of the coordination between Eu (III)/Tb (III). The O atoms in C=O, C–O (O=C–O), and –OH bonds transferred some electrons to the rare earth metals and formed Eu-O/Tb-O bond. Therefore, the electron density decreased and the binding energy of O1s increased in XPS spectra. The binding energy of C1s and O1s is summarized in Table S2 (see Supplementary Materials). The internal coordination structure of the light conversion films could be obtained combined with the FTIR analysis; two schematic diagrams of the CA-Eu structure are shown in Figure 3.

The XPS spectrum of CA-Eu film in Figure 2h revealed the characteristic peaks of Eu3d (1165.08 eV for Eu3d**_3/2_** and 1134.08 eV for Eu3d**_5/2_**), which indicated the presence of Eu (III) in CA-Eu light conversion film. These two spin orbitals made Eu^3+^ emit red and orange light, while the integration area for Eu3d**_5/2_** orbitals was larger so that the CA–Eu light conversion film mainly had red emission [23], which is consistent with the results.

**Figure 4:**
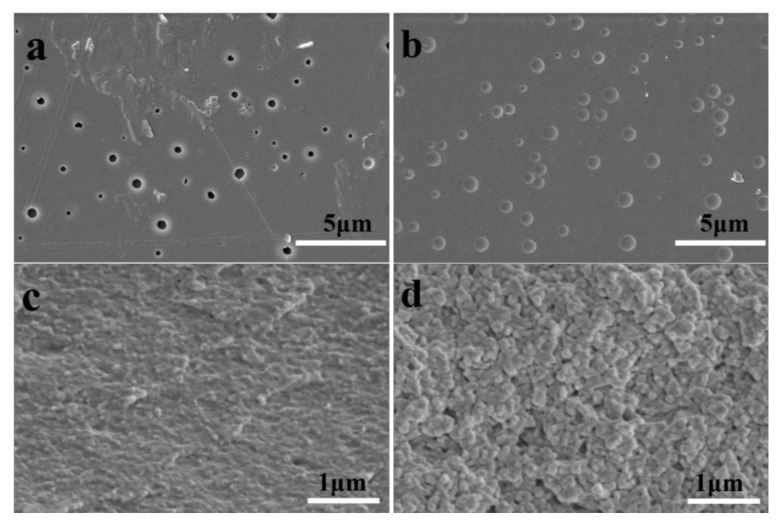
Scanning electron micrographs of CA film and CA-Eu light conversion film. (**a**) the surface of CA film, (**b**) the surface of CA-Eu film, (**c**) the section of CA film, (**d**) the section of CA-Eu film.

Figure 4 shows SEM images of the surface and section of CA film and CA–Eu film. Figure 4a,b (magnification of 10,000×) shows that the surface of the pure CA film had the porous structure of different sizes [24,25,26], however, the pores in CA–Eu light conversion film were filled. And these tiny pores at the surface are a result of the phase separation [27]. During the process of scraping the film, bubbles were generated in the film due to the rapid volatilization of acetone (act as a pore creating agent). Due to the short film formation time (about 2–3 min), the cellulose acetate solution did not have enough time to gather and formed the pores of different sizes in the bubbles. Similarly, bubbles were generated as well during preparation process of the light conversion film. However, the distance between molecules increased after doping Eu^3+^, which could reduce the entanglement between long molecular chains and accelerated the movement of molecules, thereby reducing the viscosity. The conditional viscosity was measured using the QNO-4 viscometer (CA-22.9s, CA–Eu-11.0s, it is confirmed that the viscosity reduced after doping Eu^3+^ and measurement method can be seen in Supplementary Materials.). Low viscosity made the cellulose acetate solution had enough time to gather after rapid volatilization of acetone, which shows that the pores were filled in CA–Eu film. The surface of the CA–Eu film was uniform and smooth, indicating that CA and Eu^3+^ had good compatibilities so that the high transparency of the light conversion film could be observed. Section electron micrographs (magnification of 40,000×) in Figure 4c,d show that the longitudinal distribution of the films was uniform and dense. Uniformly distributed spherical particles could be observed in the CA-Eu light conversion film. There was no obvious particle agglomeration phenomenon, further indicating that Eu^3+^ had good compatibility with CA.

**Figure 5:**
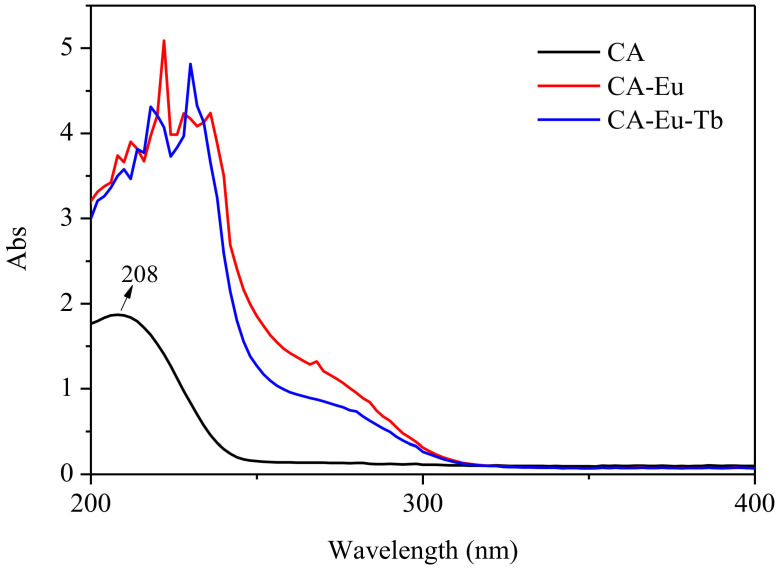
UV absorption spectra of CA, CA-Eu and CA-Eu-Tb films.

Ultraviolet absorption spectra of the pure CA and light-conversion films are shown in Figure 5. The UV absorption of the light conversion films was stronger than that of the pure CA film at 200–300 nm and converted ultraviolet light into red light. The CA film formed a broad absorption band at about 208 nm, which originated from the π–π* transition absorption peak of the carbonyl group. After doping with rare earth ions, the electronic environment around the ligand CA changed due to the presence of the central ion, which resulted in a red shift of the carbonyl absorption wavelength. Additionally, in the light-conversion films, the shape of the carbonyl absorption peak became sharper, and new absorption peaks appeared. This is mainly the result of the reaction of rare earth ions with CA to form a coordination structure. In addition, the UV absorption (300–400 nm) of the films was basically equal to zero, indicating that the light-conversion films played an important role in avoiding harmful ultraviolet radiation [28].

### 3.2. Fluorescent Characterization of the Light Conversion Films

**Figure 6:**
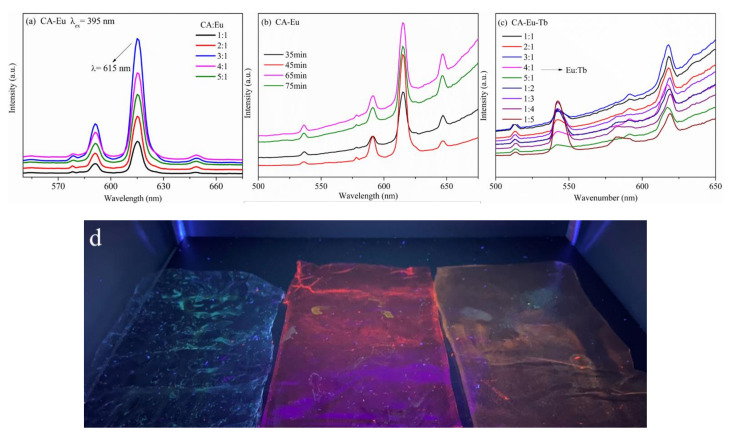
Emission spectra of light conversion films. (**a**) CA–Eu film with different proportions, (**b**) CA–Eu film with different reaction time. (**c**) CA–Eu–Tb film with different Eu^3+^:Tb^3+^ ratios, (**d**) the optical photograph of CA and the light conversion films. (From left to right are CA, CA–Eu and CA–Eu–Tb).

The emission spectra (fluorescence spectroscopy) of CA-Eu light conversion film with different proportions (λ_ex_ = 395 nm) are shown in Figure 6a. The emission light purity of CA-Eu film was high, and different ratios had no effect on the position of the emission peak. This was mainly composed of four emission peaks located at 578, 591, 615, and 649 nm, corresponding to ^5^D_0_→^7^F_0_, ^5^D_0_→^7^F_1_, ^5^D_0_→^7^F_2_, and ^5^D_0_→^7^F_3_ energy level transition of Eu^3+^. Of these, the electric dipole transition (^5^D_0_→^7^F_2_) at 615 nm made CA-Eu light conversion film emit high-purity red light; the intensity of the emission peak was the largest, indicating that the CA-Eu film could efficiently convert harmful ultraviolet light into red light beneficial to plant growth. The intensity of the magnetic dipole transition (red-orange light) at 591 nm was the second highest. Therefore, the emission peak intensity at 615 nm was used to compare the fluorescence intensity of CA-Eu light-converting films prepared under different conditions. Figure S1a (in Supplementary Materials) shows that the fluorescence intensity first increased and then decreased with increasing Eu^3+^, i.e., concentration quenching [29,30]. The initial increase in Eu^3+^ concentration implied that the number of complexes would increase accordingly; the fluorescence intensity gradually increased as well, and the coordination reaction was complete when the doping amount of Eu^3+^ exceeded a certain range; thus, the ion distance was shortened to generate energy resonance, which caused the luminescence intensity to decrease. Therefore, the fluorescence intensity of the light conversion film for a CA:Eu^3+^ ratio of 3:1 was optimal when other conditions were the same.

CA, CA-Eu and CA-Eu-Tb light conversion films were put under the ZF-20D dark-box ultraviolet analyzer (Yuhua Instrument Co., Ltd., Gongyi, China) and appeared different fluorescent color. The optical photograph was shown in Figure 6d.

Figure 6b shows the emission spectra of CA-Eu light conversion films prepared under different reaction time conditions. The intensity comparison of the characteristic emission peak of Eu^3+^ at 615 nm (red light) is shown in Figure S1b; it was obvious that the fluorescence intensity increased first and then decreased with increasing reaction time. This is because the coordination reaction was not sufficient at short times and fewer complexes were formed; thus, the fluorescence intensity was relatively low. As the reaction time continued, the coordination between CA and Eu^3+^ became increasingly complete, and more complexes were formed accordingly. As the reaction time reached 65 min, the UV absorption of the ligand was the strongest, and CA absorbed the most energy and transferred the energy to Eu^3+^ effectively at this moment; thus, optimal fluorescence properties were obtained [31]. With longer reaction times, heat would destroy the balance that established between the intra molecular and intermolecular reactions so that the length of the Eu–O bond became larger. This caused a decline in the fluorescence intensity and proved that the Eu–O bond distance had an important effect on the emission intensity: A shorter bond length leads to a higher luminescence intensity [26]. Therefore, the fluorescence intensity of the light conversion film for the reaction time at 65 min was optimal when other conditions were the same.

The emission spectra of CA–Eu–Tb light conversion films with different Eu/Tb doping ratios (the total amount of rare earth ions was fixed) are shown in Figure 6c. In addition to the emission peak of Eu^3+^ in the fluorescence spectrum, the characteristic emission peaks of Tb^3+^ could also be monitored under excitation light at 375 nm (the characteristic excitation wavelength of Tb^3+^) located at 488, 542, 583, and 620 nm corresponding to ^5^D_4_→^7^F_6_, ^5^D_4_→^7^F_5_, ^5^D_4_→^7^F_4_ and ^5^D_4_→^7^F_3_ energy level transitions in Tb^3+^, respectively. Figure S1(c) compares the intensity of the characteristic emission peak at 615 nm. The fluorescence intensity of CA-Eu-Tb light-conversion film was stronger than that of CA–Eu light conversion film (Eu:Tb = 1:0), which indicated that Tb^3+^ could sensitize Eu^3+^ to emit light. After the CA–Eu–Tb film absorbed the ultraviolet light with a wavelength at 375 nm, part of the energy was transferred to the ^5^D_4_ energy level of Tb^3+^; the other part of the energy was absorbed by Eu^3+^, making it transition from the ground state (^7^F_0_) to the excited state (^5^L_6_). Part of Tb^3+^ at the ^5^D_4_ energy level transitioned to the ^7^F_5_ excited state to generate visible light at 545 nm—most of that was transferred to the ^5^D_1_ energy level of Eu^3+^ and then transitioned to the ^5^D_0_ energy level through cross relaxation. Eu^3+^ at the ^5^L_6_ energy level reached the ^5^D_0_ energy level after (^5^D_3_, ^5^D_2_, ^5^D_1_) continuous cross relaxation, and then transitioned to (^7^F_1_, ^7^F_2_, ^7^F_3_) energy level to generate the light with different wavelengths and realize the energy transfer between Tb^3+^ and Eu^3+^. This process increased the luminescence intensity of Eu^3+^ and played a sensitization role [32]. In addition, the fluorescence intensity increased first and then decreased with increasing Tb^3+^ concentration, which led to concentration quenching. The fluorescence performance of the light conversion film was optimal at a Eu^3+^:Tb^3+^ ratio of 3:1 (0.75:0.25).

### 3.3. Performance Characterization of the Films

**Figure 7:**
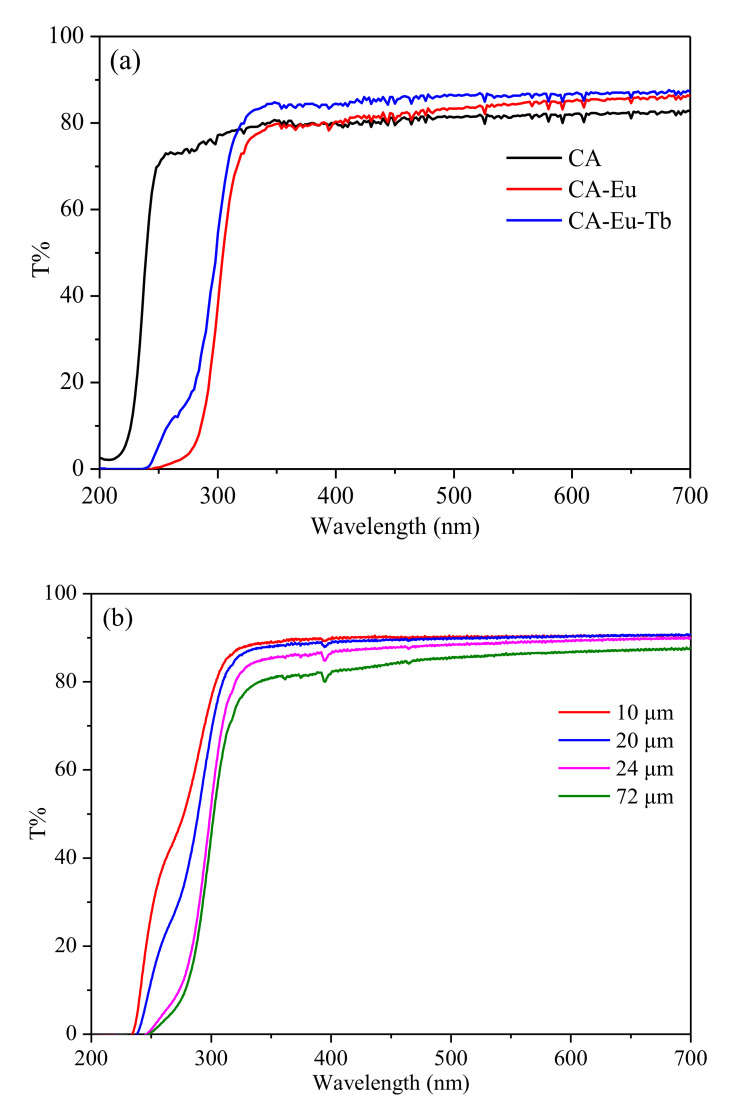
UV-visible transmittance spectra of CA, CA–Eu and CA–Eu–Tb films. (**a**) the transmittance spectra, (**b**) the transmittance spectra of CA–Eu film of different thicknesses.

Figure 7 shows the UV-visible transmission spectra of the films at 200–700 nm. The pure CA film had good UV-Vis transmittance (80%). The light transmittance from 200 to 240 nm was almost 0%, but it reached ~80% after 240 nm; CA–Eu–Tb film had the strongest light transmittance and reached 86%. The pores probably scatter the light, and the film with greater porosity makes higher scattering intensity [33]. SEM images show that CA film had the porous structure and the pores were filled in CA–Eu film, so the light transmittance of the light conversion films was higher than that of the CA film. On the other hand, the section electron micrograph of CA film shows that the longitudinal distribution was uniform and dense and the molecular was the stretched state, which indicated the light transmission probably reduced. After doping Eu^3+^, the stretched molecular changed into uniformly distributed spherical particles structure in CA–Eu film, which may leave the empty space and conducive to light transmission. Therefore, the light transmittance of the light conversion films was higher than that of the CA film. The average light transmittance of the films at 200–350 nm and 350–700 nm is summarized in Table 1. Compared with the pure CA film, the light conversion films had a significant effect on UV absorption and could maintain a high transmittance of the CA film as well.

Adjusting the size (250–1000 μm) of the preparation device could lead to different thicknesses of light conversion films. A stainless steel electronic digital caliper (Shenhan Measuring Tool Co., Ltd., Shanghai, China) was used to accurately measure the thickness of the films. Five points were selected on the films to measure and take the average values, and the actual thicknesses could be obtained: 1000–72 μm, 750–24 μm, 500–20 μm, and 250–10 μm. Figure 7b shows the UV-visible transmission spectra of CA-Eu light conversion films for different thicknesses and the average light transmittance in the wavelength range of 200–700 nm was calculated in Table 2. Obviously, as the film thickness decreased, the transmittance of CA–Eu film in the visible light region gradually increased. The average light transmittance of CA–Eu light conversion film with a thickness of 10 μm could reach the maximum exceeding 90%. Compared with the previously work, the light conversion films present the high transmittance in the visible range [34,35,36,37].

**Figure 8:**
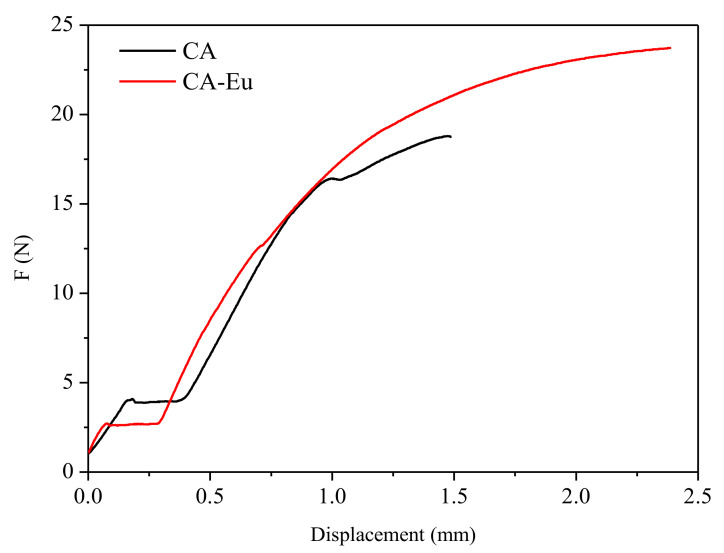
The load-displacement curves of CA and CA–Eu films.

Mechanical properties include tensile strength that represents the strength, and elongation at breaking that reflects flexibility and elasticity of the films. The tensile strength could be calculated by the formula:

(1) $$\sigma_{M}=\frac{F}{b\times d}$$

where σ_M_ represents the tensile strength (MPa), F is the maximum tensile force (N), b is the width (mm), and d is the thickness (mm) of the films. The elongation at breaking could be calculated by the formula:

(2) $$\varepsilon_{B}=\frac{L-L_{0}}{L_{0}}\times 100\%$$

where ε_B_ is the elongation at breaking (%), L_0_ is the distance between the clamps before the film was stretched (mm), and L is the distance between the clamps when the film was broken (mm). Figure 8 shows the load-displacement curves of CA and CA–Eu films; the mechanical properties are summarized in Table 3. The tensile strength and elongation at breaking of CA films were 11.4 MPa, and 2.7%, respectively. The mechanical properties of CA–Eu light conversion film were higher than those of CA film, which were 13.3 MPa, and 4.8%, respectively. These results indicated that the tensile strength of CA film could be enhanced by adding Eu^3+^ because of the interaction between Eu^3+^ and CA molecules to form a stable coordination structure. The interaction also enhanced the fluidity of the molecular chain to enhance the flexibility and elasticity of the light transfer films on the macroscale [27,38]. And CA–Eu light conversion film presents good mechanical properties compare with the previously reported CA based films, such as cellulose acetate–polyurethane (CA–PU) film [39], the CA-based composite membranes incorporated with bamboo-based lignocellulose nanofibrils (LCNFs) [40] and et al.

**Figure 9:**
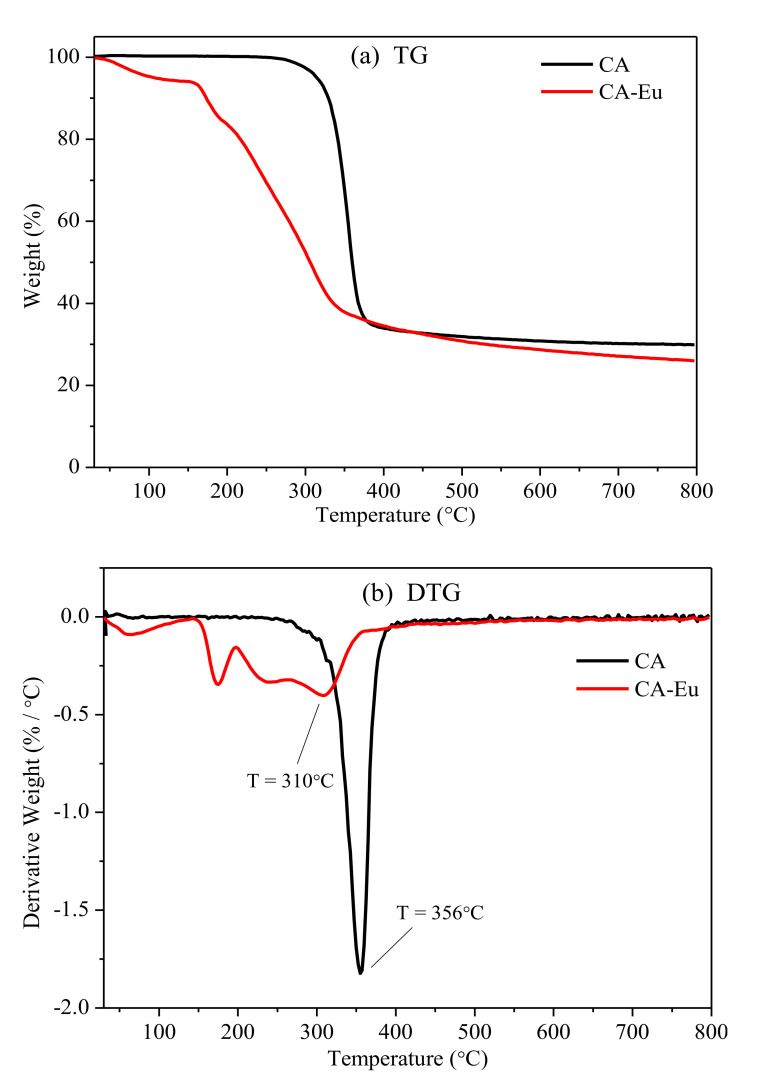
Thermogravimetric (TG) and derivative thermogravimetric (DTG) curves of CA and CA-Eu films. (**a**) TG, (**b**) DTG.

The thermogravimetric (TG) and derivative thermogravimetric (DTG) curves of CA and CA–Eu films are shown in Figure 9. The TG curve showed that the quality of the pure CA film remained basically unchanged before 250 °C, and the acetyl group on the CA molecule chains began to undergo pyrolysis when the temperature rose to 250 °C. The acidity generated by the dissociation of the acetyl group would accelerate the degradation of CA molecule chains so that the weight loss rate gradually increased. The DTG curve indicates that the largest weight loss rate of CA film was at 356 °C. The weight loss rate then gradually decreased, and the quality was basically stable above 410 °C. Finally, the residual mass of the CA film was 29.94%. The initial weight loss temperature and residual mass were relatively lower for the CA–Eu film, indicating that its thermal stability was poor. There were three obvious weight loss stages: (1) Evaporation of trace residual solvent and adsorbed water between 40 °C and 143 °C [41], where the weight loss was less about 5.94%; (2) 143–194 °C due to the pyrolysis of Eu–O coordination structure and heat loss of 9.63%; and (3) main mass loss above 194 °C. The final stage was mainly the degradation and carbonization of the CA molecular backbone [42]; the weight loss rate reached a maximum at 356 °C and then gradually decreased. Until 544 °C, the weight of CA-Eu film basically no longer changed and finally the residual mass was 26.02%.

**Figure 10:**
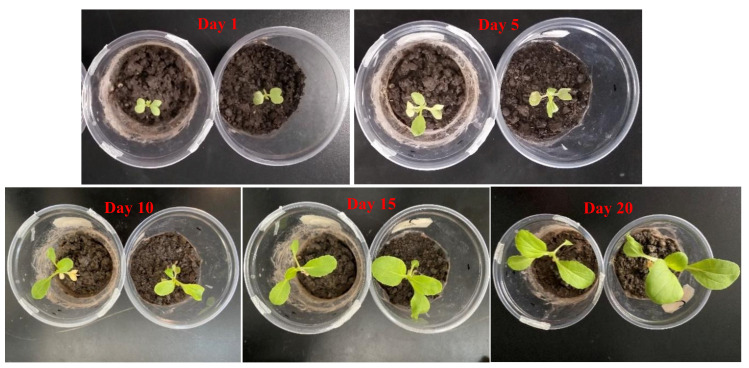
The growth of cabbage seedlings from day 1 to day 20. (**left**—CA film, **right**—CA-Eu film).

**Figure 11:**
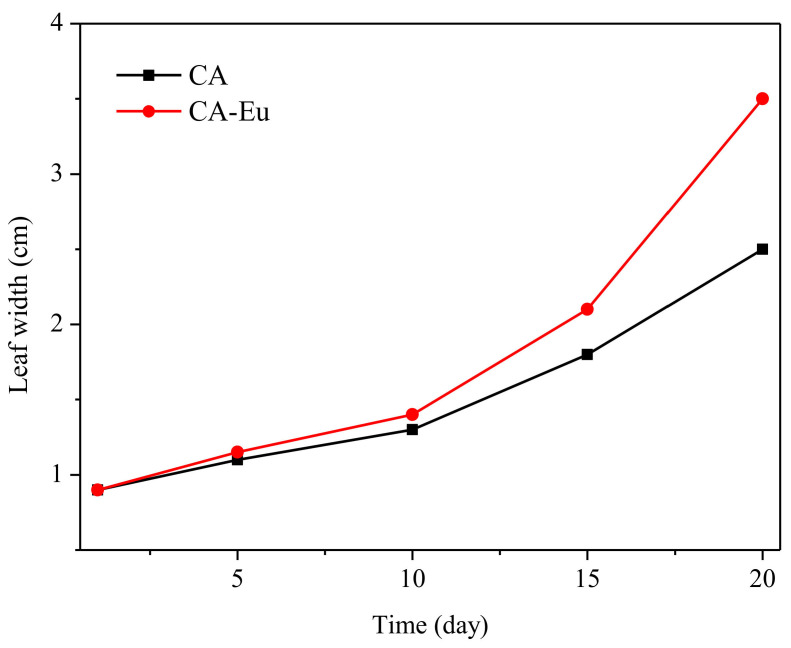
Change of maximum leaf width from day 1 to day 20.

Cabbage seeds were grown in the soil. The seedlings were then covered with CA film and the CA–Eu light conversion film and placed in an environment with the same temperature, light, and moisture conditions for 20 days [43]. Figure 10 recorded the growth of seedlings from day 1 to day 20. The maximum leaf width changes are shown in Figure 11. The growth rate of the two seedlings in the first 10 days was almost the same. The growth rate of the seedlings increased, and it was obvious that the growth of the seedling covered by the CA–Eu light conversion film was better than that of CA film. The seedlings covered by a CA–Eu film had a larger leaf width (CA film: 2.5 cm; CA–Eu film: 3.5 cm). They also had more leaves and were higher, which proved that CA–Eu light conversion film could indeed convert ultraviolet light into red light for a positive effect on the photosynthetic growth of plants.

## 4. Conclusions

(1) Eu^3+^ reacted with CA and formed an Eu-O coordination bond with the O atoms in C=O, C–O (O=C–O), and O–H of CA structure. The organic ligand CA has a good absorption coefficient at UV wavelength range so that it could effectively transfer energy to Eu^3+^ through ultraviolet absorption and make CA–Eu light conversion film emit high-purity red light through ^5^D_0_→^7^F_2_ electric dipole transition. Due to the concentration quenching effect, the optimal fluorescence intensity was obtained at a CA: Eu^3+^ ratio of 3:1. In addition, the influence of the reaction time was also explored and the optimal fluorescence performance was obtained for the reaction time at 65 min.
(2) After doping Tb^3+^ in the light-conversion films, the Tb^3+^ could effectively increase the fluorescence intensity of Eu^3+^ to play a sensitization role in the process of energy transfer.
(3) The prepared light conversion film had a simple preparation process, high transparency, high tensile strength, and good flexibility. The material could absorb ultraviolet light and convert it into red light beneficial for the growth of plant photosynthesis. The material was also biodegradable, which is useful for efficiency and environmental protection.

## Figures and Tables

**Table 1:** Mean of the pure CA and light-conversion film for transmittance.

Sample	T% Mean(200–350 nm)	T% Mean(350–700 nm)
CA	54.3625	81.20739
CA–Eu	24.0450	83.23807
CA–Eu–Tb	30.5563	85.94716

The above data is calculated by origin 8.5. (OriginLab, USA)

**Table 2:** Mean of light-conversion film (CA–Eu) of different thicknesses for transmittance.

Sample (CA–Eu)	T% Mean(200–350 nm)	T% Mean(350–700 nm)
10 μm	43.78562	90.17846
20 μm	37.51844	89.81298
24 μm	29.26031	88.35093
72 μm	26.37656	85.15506

**Table 3:** The mechanical properties of CA film and CA-Eu light conversion film.

Sample	σ_M_ (MPa)					Mean/SD	ε_B_ (%)					Mean/SD
	1	2	3	4	5		1	2	3	4	5	
CA	6.3744	13.0921	13.8606	12.2708	11.5697	11.4/3.0	2.8119	3.3088	1.8889	2.3977	3.2536	2.7/0.6
CA-Eu	16.2126	13.1485	9.6287	14.9523	12.7006	13.3/2.5	5.9627	5.309	4.9064	4.1741	3.8461	4.8/0.9

SD represents the standard deviation.

## Data Availability

The data presented in this study are available in the insert article.

## Supplementary Materials

The following are available online at https://www.mdpi.com/2073-4360/13/1/113/s1, Figure S1: Fluorescence intensity of the light conversion films at the peak of 615 nm, Table S1: The characteristic FTIR data of the pure CA and light conversion films, Table S2: Binding energy of C1s and O1s for CA and light-conversion films, Table S3: The conditional viscosity of CA and CA-Eu solutions.
