# Supplementary material for: One-Step Synthesis of Eu3+-Modified Cellulose Acetate Film and Light Conversion Mechanism
Source: Polymers (Basel). 2020 Dec 30;13(1):113. doi: 10.3390/polym13010113 (PMC7795846; doi:10.3390/polym13010113)
Supplement: Supplementary file 1 [file polymers-13-00113-s001.pdf]

Table S1. The characteristic FTIR data of the pure CA and light conversion films.

**Sample**	**ν-O-H**	**Δ**	**ν_{as}-COO^-**	**Δ**	**ν-COO^-**	**Δ**	**ν_{as}-C-O-C**	**Δ**	**ν-C-O-C**	**Δ**
CA	3480	0	1431	0	1737	0	1215	0	1031	0
CA-Eu	3396	-84	1475	+44	1721	-16	1225	+10	1027	-4
CA-Tb	3396	-84	1481	+50	1720	-17	1231	+16	1027	-4
CA-Eu-Tb	3396	-84	1479	+48	1718	-19	1231	+16	1027	-4

Table S2. Binding energy of C1s and O1s for CA and light-conversion films.

**Sample**	**C1s**			**O1s**		
	**C-C (C-H)**	**C-O**	**C=O**	**-OH**	**C-O**	**C=O**
CA	284.8	286.774	289.002	531.493	531.907	532.693
CA-Eu	284.8	286.771	288.839	531.945	532.393	533.131
CA-Eu-Tb	284.8	286.730	288.733	531.740	532.297	533.060

The above data is calculated by CasaXPS.

Measurement method of the conditional viscosity: The conditional viscosity was measured using the QNO-4 viscometer (Material Testing Machine Factory, Tianjin, China) and the test method was based on GB/T 1723-93, and five sets of films were tested in parallel. The conditional viscosity of CA and CA-Eu solutions was summarized in Table S3.

Table S3. The conditional viscosity of CA and CA-Eu solutions.

**Sample**	**Conditional viscosity (s)**					**Mean**
	**1**	**2**	**3**	**4**	**5**	
CA	21.34	22.21	22.22	24.01	24.91	22.9
CA-Eu	8.75	10.00	11.45	11.84	13.20	11.0

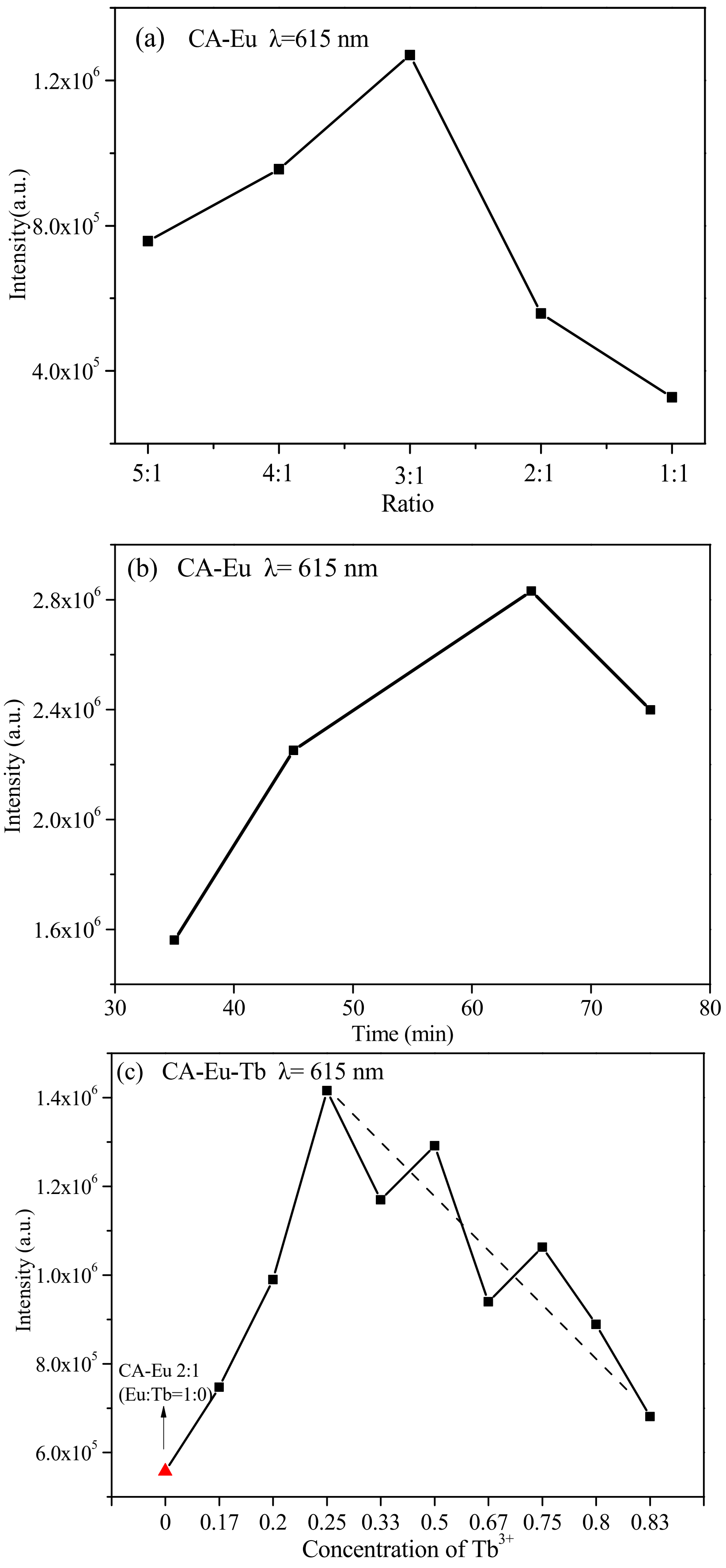

Figure S1. Fluorescence intensity of the light conversion films at the peak of 615 nm. a–the intensity of different proportions. b–the intensity of different reaction time. c–the intensity of different Eu^{3+}:Tb^{3+}

ratios. (For convenience, set the total amount of Eu^{3+} and Tb^{3+} to "1" and calculate the proportion of Tb^{3+} proportionally. And use the proportion of Tb^{3+} as the abscissa and the fluorescence intensity at 615 nm as the ordinate to plot Figure S(c).)
